# Proximal Fibula Flap for Upper Extremity Reconstruction in Pediatric Patients: A Multicentric Study

**DOI:** 10.7759/cureus.105998

**Published:** 2026-03-27

**Authors:** Muhammad Talha Khan Lodhi, Muhammad Omar Afzal, Hafiz Usman Arshad, Muhammad Hamza Naeem Jan, Kamran Khalid, Taha Ahmad, Ilyas Rafi

**Affiliations:** 1 Plastic and Reconstructive Surgery, Shaukat Khanum Memorial Cancer Hospital and Research Centre, Lahore, PAK; 2 Orthopedic Surgery, Shaukat Khanum Memorial Cancer Hospital and Research Centre, Lahore, PAK; 3 Plastic and Reconstructive Surgery, Jinnah Burn and Reconstructive Surgery Centre, Lahore, PAK; 4 General Surgery, Shaukat Khanum Memorial Cancer Hospital and Research Centre, Lahore, PAK; 5 Surgical Oncology, Shaukat Khanum Memorial Cancer Hospital and Research Centre, Lahore, PAK

**Keywords:** bone transplantation, free fibula flap, free tissue flaps, microsurgery free flaps, oncology pediatrics

## Abstract

Background: Functional reconstruction to restore limb length of the upper limbs after sarcoma resection is always challenging for reconstructive surgeons, particularly in pediatric patients, keeping in mind the necessity of matching the growth of limbs. We present our cases of using vascularized proximal fibula free flap, along with physis based on anterior tibial vessels, for upper limb reconstruction.

Methods: In this study, we conducted a retrospective analysis of 14 consecutive patients who underwent vascularized proximal fibula flap transfer for autologous functional reconstruction following oncologic resection of the humerus between April 2022 and April 2024. We evaluated all patients preoperatively and postoperatively, documenting outcomes like an increase in bone length, bony union, and donor site morbidity.

Results: A total of 14 patients with an average age of 9.0 ± 2.4 years were included in this study. The average length of the humerus defect after surgical resection was 14.4 ± 2.3 cm. All patients had a smooth postoperative period with no wound healing issues and 100% flap survival rate. There was a measurable increase in bone flap length with complete bone union in all cases. One patient experienced brief common peroneal nerve palsy postoperatively. In none of the cases was knee instability noted. The mean follow-up period was 17.4 ± 2.6 months. The mean Musculoskeletal Tumor Society (MSTS) score was 29.

Conclusion: Vascularized proximal fibula free flap based on anterior tibial vessels not only restores immediate limb length, but the growth allows long-term increase in limb length with minimal donor site morbidity.

## Introduction

The proximal humerus is a common location for primary bone tumors in the pediatric population and accounts for about 10-15% of all primary malignant bone tumors, also making it the third most common site for primary bone tumors after the distal femur and proximal tibia [1]. Malignant bone tumors account for around 5% of all malignant neoplasms in children [2]. Osteosarcoma and Ewing’s sarcoma are the two most common malignant bone tumors in children, causing a significant threat to their lives [3,4].

During recent times, advances in neoadjuvant chemotherapy and radiation therapy have increased the survival rates of the patients, but surgical excision of the tumor remains the mainstay of treatment to provide local control of the disease, and limb salvage surgeries have decreased the ratio of amputation as well [5-8].

Resection of bony tumors often results in substantial bone defects, shorter limbs, and shoulder joint integrity disruption as well [9]. Patients with primary bone tumors are quite young and have higher functional demands, so they need durable and long-term reconstruction. The reconstruction should provide long-term stability, allow early range of motion and early initiation of function at the elbow and hand, and restore limb length. Restoring limb length to prevent limb length discrepancy is most important as it can cause a negative impact on the patient’s self-confidence and decrease long-term functional outcomes as well [10].

Prosthetic replacement is used most commonly for bony replacement; however, it is not favorable in children as it is associated with implant fractures, and the most critical factor remains the bony growth development in children during their growing age [11].

For segmental defects, vascularized fibula bone flap remains the gold standard for upper extremity reconstruction. Vascularized proximal fibula free flap based on anterior tibial vessels allows early union through primary bone healing into recipient site, remodeling and early weight baring, good toleration of postoperative radiation, and reduction of the risk of subsequent fractures [12], and if transferred with proximal physis it allows longitudinal limb growth and hypertrophy and decreases the chances of limb length discrepancy making it a best option for humerus reconstruction.

Despite the established role of vascularized fibula flaps in long bone reconstruction, there remains limited data specifically evaluating the outcomes of proximal fibula free flap with physeal transfer for humeral reconstruction in pediatric patients, particularly in those receiving postoperative radiotherapy. In this study, we retrospectively analyzed a case series involving pediatric patients who underwent vascularized fibula flaps for humerus reconstruction following oncologic resection of the bony tumors at our institution. Our objective was to evaluate the outcomes, complications, and survival rates associated with this surgical procedure and postoperative radiation. 

This article was previously presented as a poster presentation at the 2025 Shaukat Khanum Cancer Symposium on October 24, 2025. 

## Materials and methods

This was a retrospective cohort study through chart review of all patients who underwent humerus reconstruction using free vascularized fibula flap done at Shaukat Khanum Memorial Cancer Hospital & Research Centre, Lahore, Pakistan, and Jinnah Burn and Reconstructive Surgery Centre, Lahore, Pakistan, between April 2022 and April 2024. Informed Consent was taken from all patients and their guardians. The study was granted exemption by the Shaukat Khanum Memorial Trust Institutional Review Board (EX-14-05-25-01).

Study population

Patients aged less than 18 years, with osteosarcoma or Ewing's sarcoma of the proximal humerus involving the head, undergoing vascularized fibula free flap for reconstruction, and with adequate donor site vascularity confirmed by preoperative imaging were included in the study. Exclusion criteria included: patients with previous Injury to the donor site, surgery, or pathology involving the proximal fibula region.

Outcomes

The primary outcome of this study was flap survival and successful reconstruction of the humeral defect following tumor resection using a vascularized proximal fibula free flap. Secondary outcomes included bony union, increase in bone length during follow-up, postoperative complications, donor site morbidity, and functional outcomes measured using the Musculoskeletal Tumor Society (MSTS) score [13].

Study process, definitions, and measurements

Preoperative contrast-enhanced magnetic resonance imaging (MRI) of the humerus, contrast-enhanced computerized tomography (CT) of the chest, abdomen, and pelvis, and bone scans were performed to assess the size of the tumor, its extent, and relationship with neighboring neurovascular bundle to determine the outcomes of surgery and check for distant metastasis and skip lesions to exclude the contraindications of limb salvage.

Bony union was defined radiologically as the presence of bridging callus across at least three of four cortices on orthogonal radiographs, along with the absence of pain or tenderness at the graft-host junction on clinical examination. Increase in bone length was measured using serial standardized radiographs by calculating the difference in length of the reconstructed humerus from fixed anatomical landmarks over time. Measurements were performed using digital radiographic software. Functional outcomes were assessed using the MSTS scoring system. All evaluations were performed by the treating surgical team; however, due to the retrospective nature of the study, blinding was not feasible.

Demographic and clinical data, including patient age, gender, tumor type, bone defect length, surgical details, and postoperative outcomes, were collected from electronic medical records. All data were analyzed using descriptive statistics due to the small sample size. 

Vascular anatomy and surgical technique

There is an anastomotic vascular network supplying the proximal fibula [14]. It is formed superiorly by the inferior lateral genicular artery and inferiorly by the anterior tibial artery. The lateral genicular artery arises from the popliteal artery posteriorly and moves inferiorly and laterally to wrap around the head of the fibula. The recurrent anterior tibial artery is the first branch after the bifurcation of the popliteal artery into the anterior and posterior tibial arteries. It is given off from the anterior tibial artery just as it pierces the intermuscular septum and comes anteriorly to supply the fibular physis. Proximal fibula physeal transfer can be transferred using the inferior lateral genicular or anterior tibial artery as the physis is not supplied by peroneal vessels before the growth ceases [15].

Preoperative CT angiogram is always performed, which enables us to understand the vascularity more clearly. The patient can be positioned into the lateral decubitus or supine position. We start with the inverted J-shaped skin incision extending across the neck of the fibula and vertically over the anterior compartment till 6 cm above the lateral malleolus, under tourniquet control.

**Figure 1:**
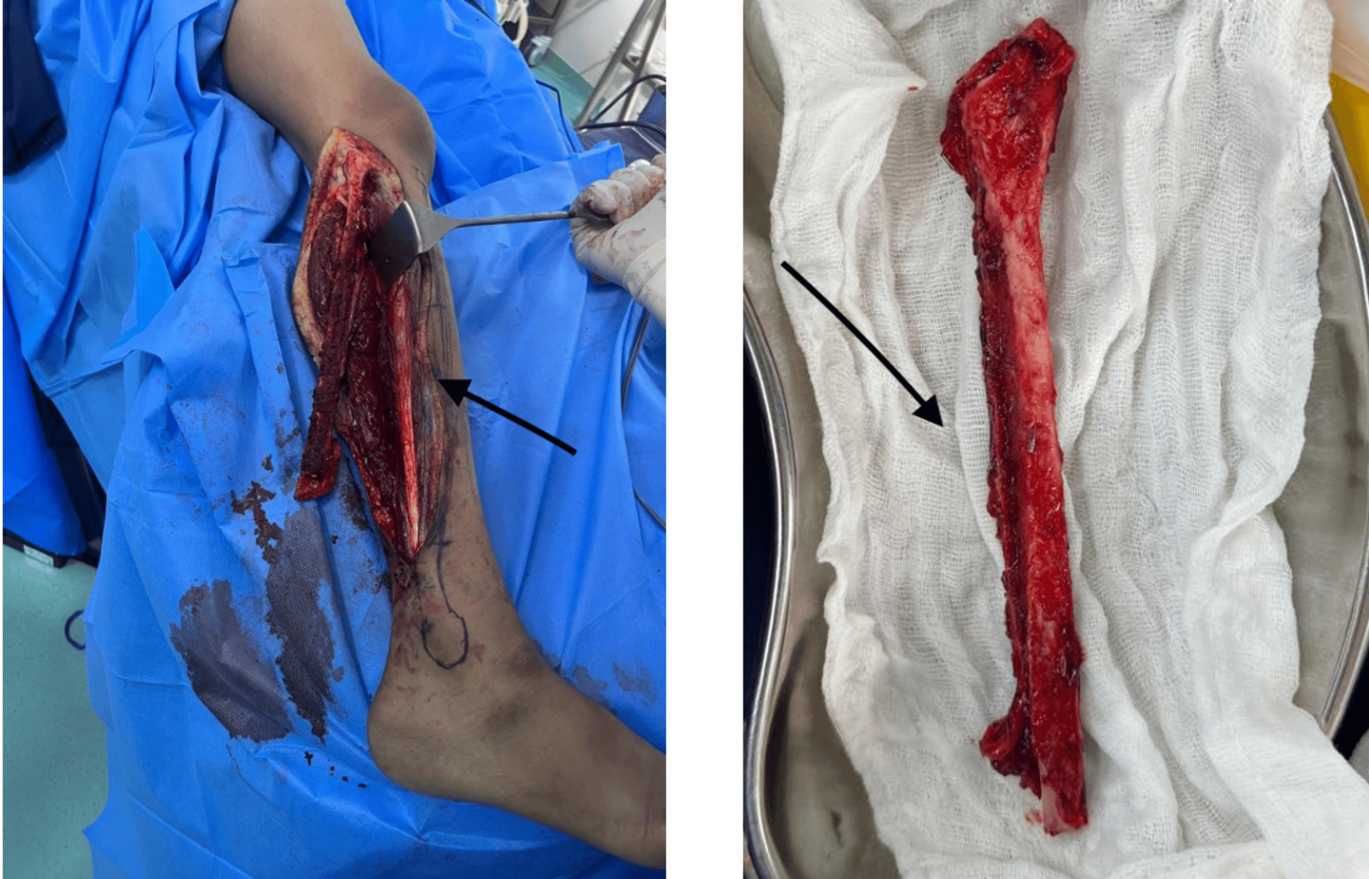
Harvested free fibular graft on anterior tibial vessels (black arrow)

Dissection is done to separate the septum between the tibialis anterior and extensor digitorum longus and extensor hallucis, which gives access to the anterior tibial vessels. More dissection is performed to preserve the anterior tibial vessel for flap vascularity. All the medial perforators from the vessels are clipped, and lateral perforators that are deep and are musculoperiosteal are preserved. Dissection is performed, leaving behind the cuff of muscles and intact periosteum to preserve the vascular supply to the fibula. The peroneal muscles are dissected off from the fibula, leaving the cuff of muscle and periosteum intact. Distal osteotomy is performed first. The proximal 2/3rd of the fibula can be harvested on the anterior tibial vessels [16-18], as shown in Figure 1.

Proximally, medial branches of the common peroneal nerve supplying tibialis anterior are preserved, especially the most proximal branch, which branches out right where the peroneal nerve loops around the neck of the fibula. Similarly, all branches of the peroneal nerve to the extensor hallucis and extensor digitorum longus are preserved by dissecting off the vessels. Minimal handling of the nerve ensures the prevention of postoperative neuropraxia. Incision is given at the neck of the fibula, and the nerve is traced inferiorly and separated. More proximally, the muscle cuff above the neck of the fibula is preserved over the head, as it contains the branch supplying the fibula physis. The head is then disarticulated with the slip of the biceps femoris tendon so that it can be anchored at the recipient site. The lateral collateral ligament is divided as close to the head as possible so that it can be attached to the tibial condyle later on. The tourniquet is released, and the vascularity of the flap is confirmed. Adequate length of the anterior tibial vessel can be harvested with the bone. Once vascularity is confirmed, the fibula is gently pulled with traction, and dissection is done to gain as much length of the anterior tibial as possible proximal to the physis branch, which usually comes out as the vessels come into the anterior compartment. Up to 2 cm of proximal length can be gained in pediatric patients, which can facilitate antegrade anastomosis if necessary. After preparation of the recipient site, the vessels are divided.

The detached fibula and fibular physis, along with its vascular supply, are transferred to the recipient bed. The shaft of the fibula is telescoped 1 cm into the medullary canal of the remaining humerus and fixed with a dynamic compression plate. The fibular head articular surface is then aligned with the glenoid cavity. The rotator cuff or the remaining capsule is placed over the fibular head and stitched with non-absorbable suture, without compromising the physis branch. A portion of the lateral collateral ligament is used to reinforce the repair.

Microvascular anastomosis of the anterior tibial vessels is performed distally, with the brachial vessels in an end-to-side fashion. Closure is done, and the drain is placed. The donor site is closed after securing hemostasis and drain placement. An implantable Doppler is placed to monitor the integrity of the vascular anastomosis.

## Results

We analyzed 14 cases in which a proximal free fibula flap was used to reconstruct humeral defects. Of them, 10 were male patients; 13 cases involved proximal humeral defects, and one case involved a distal humeral defect. In 13 cases, the fibula flap was transferred alone, while in one case, a concomitant latissimus dorsi flap was used to restore elbow flexion. The mean age of patients was 9.0 ± 2.4 years. The mean follow-up period was 17.4 ± 2.6 months (range, 12-20 months). All proximal fibula flaps were performed with retrograde anastomosis.

All free flaps survived without any vascular complications. No cases demonstrated wound breakdown, hematoma formation, or donor site vascular compromise. The average increase in bone flap length at one year was 1.4 cm, with a maximum gain corresponding to the case with the longest follow-up. Bony union was achieved in all cases. There were no instances of flap fracture, knee instability, or tibial bowing. In one case, a nerve branch to the tibialis anterior had to be repaired after being divided during flap harvest; the patient regained full foot dorsiflexion by six months.

All sarcoma patients received postoperative chemotherapy and/or radiotherapy. The mean MSTS score was 29/30, indicating excellent function. Pain, emotional acceptance, hand position, and manual dexterity each scored 5 points in all patients. Functional scores ranged from 3 to 5 points, while lifting ability ranged from 4 to 5 points.

Representative case

**Figure 2:**
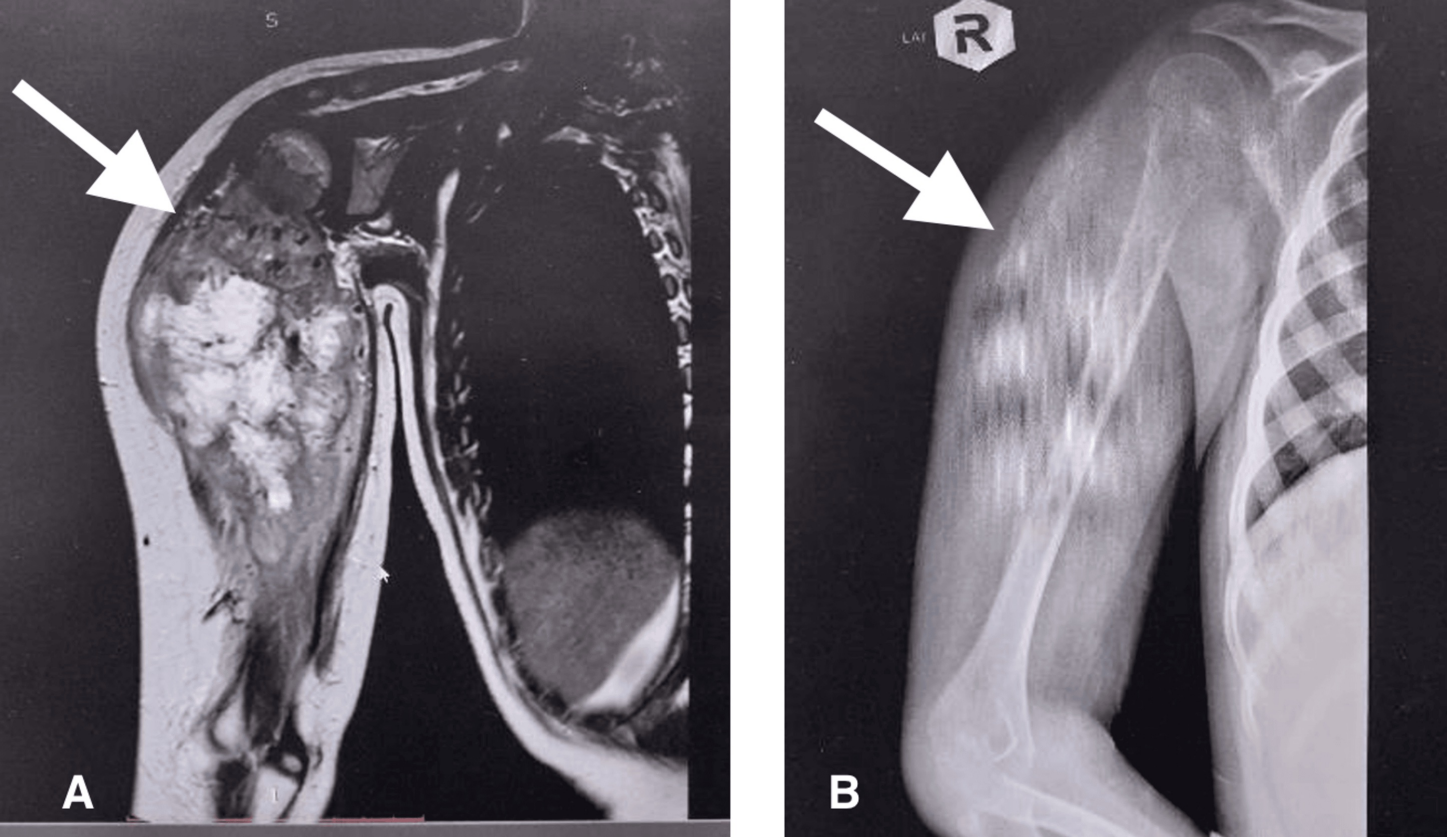
Preoperative (A) MRI T2-weighted image (coronal view) and (B) X-ray of a 14-year-old male patient showing the tumour (white arrow)

Preoperative radiograph of a 14-year-old male patient presenting with osteosarcoma involving the humerus is shown in Figure 2.

**Figure 3:**
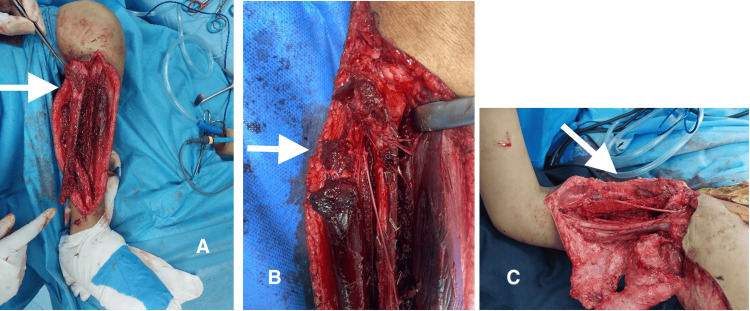
Intraoperative images showing donor site and recipient site (A) Free fibula flap based on anterior tibial vessels (white arrow); (B) Branch to head of fibula was preserved (white arrow); (C) Free fibula flap placed in the recipient site (white arrow)

Intraoperative photograph (Figure 3) following tumor resection shows the harvesting of a free proximal fibula flap based on the anterior tibial vessels, with preservation of the branch to the fibular head. The flap was anastomosed to the brachial artery in an end-to-side fashion.

**Figure 4:**
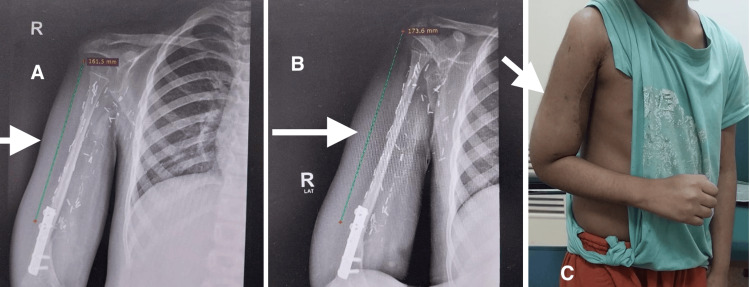
X-rays and cinical image of the patient at the 12-month follow-up (A) X-ray (anterioposterior view) showing adequate fixation; (B) X-ray (lateral view); (C) Patient's reconstructed limb after 12 months

Follow-up radiographs were done as shown in Figure 4, demonstrating successful incorporation of the graft with evident longitudinal growth and an increase in bone length.

## Discussion

This study demonstrates that the vascularized proximal fibula free flap with physis transfer is a reliable and effective method for humeral reconstruction in pediatric patients following oncologic resection, specifically in those patients in whom postoperative radiation is to be given. Importantly, our series reported 100% flap survival with minimal complications, reinforcing the safety of this technique.

Fibular physis transfer based on the anterior tibial artery provides the advantage of large-calibre vessels. Reverse vascular anastomoses were performed between the distal segment of the anterior tibial vessels and the brachial artery and accompanying veins. This approach resulted in a vascular pedicle of adequate length and offered a sufficiently wide operative field, thereby facilitating microsurgical handling [19].

Our findings align with prior studies that have established the fibula flap as the gold standard for long bone reconstruction in children due to its ability to integrate, remodel, and tolerate adjuvant therapy. In our series, the complication rate was very low, with only one transient case of common peroneal nerve palsy, which is consistent with previously reported donor-site morbidity. We acknowledge that our study included a relatively small, consecutive cohort, which may limit the generalizability of these results and could contribute to the lower complication rates observed. Additionally, all procedures were performed by an experienced microsurgical team, and the surgeon's expertise may have significantly influenced outcomes.

Our findings are comparable to those reported by Arif et al., who evaluated vascularized free fibula flap reconstruction for pediatric long bone sarcomas in a retrospective series of 10 patients [20]. In their study, osteosarcoma was the most common tumor (70%), and the humerus was involved in 40% of cases. Similar to our findings, they reported a 100% flap survival rate, demonstrating the reliability of vascularized fibula flaps for limb salvage reconstruction.

Functionally, the mean MSTS score of 29 is comparable to the other published reports. Previous studies described good outcomes in pediatric patients, although flap fractures and delayed union were reported in their cohort. In contrast, none of our patients experienced fracture or delayed union, possibly due to careful surgical technique and robust postoperative monitoring. Another critical observation is the capacity for longitudinal growth and hypertrophy of the fibula bone, and the ability to tolerate postoperative radiation therapy when the proximal physis is included. This is particularly advantageous in pediatric patients, helping to minimize limb length discrepancy and preserve long-term function.

Compared with prosthetic reconstruction, which carries a high risk of implant failure and does not accommodate skeletal growth, the vascularized fibula free flap with physis based on the anterior tibial vessel offers a biological, durable solution that adapts to the child’s development. This makes it especially valuable in young patients, where future growth is expected.

This study has several limitations. First, the study was conducted at a limited number of institutions with a relatively small sample size, which may limit the generalizability of the findings. Second, the retrospective design of the study may introduce selection and information bias. Third, the follow-up period was relatively short to fully evaluate long-term functional outcomes, continued skeletal growth, and potential late complications such as graft fracture or limb length discrepancy. Larger multicenter studies with longer follow-up are required to better evaluate the long-term durability and functional outcomes of vascularized proximal fibula transfer with physeal preservation in pediatric humeral reconstruction.

## Conclusions

Vascularized proximal fibula free flap transfer with preservation of the physis based on the anterior tibial vessels is a reliable reconstructive option for pediatric humeral defects following tumor resection. This technique provides a durable biological reconstruction with excellent flap survival, good functional outcomes, and minimal complications. Importantly, preservation of the proximal fibular physis allows continued longitudinal bone growth, helping to reduce limb length discrepancy in skeletally immature patients. Therefore, this approach represents an effective limb-salvage strategy for children undergoing resection of malignant humoral tumors.
